# Long-term effects of motivational interviewing vs. traditional counseling on dog owners’ adherence to veterinary dental home care: a three-year follow-up study

**DOI:** 10.3389/fvets.2024.1296618

**Published:** 2024-02-26

**Authors:** Karolina Brunius Enlund, Birgitta Jönsson, Kajsa H. Abrahamsson, Ann Pettersson

**Affiliations:** ^1^Department of Clinical Sciences, Swedish University of Agricultural Sciences, Uppsala, Sweden; ^2^Department of Periodontology, Institute of Odontology, The Sahlgrenska Academy, University of Gothenburg, Gothenburg, Sweden; ^3^The Public Dental Health Service Competence Centre of Northern Norway, Tromsø, Norway

**Keywords:** veterinary communication, compliance, tooth brushing, veterinary dentistry, dental health, dental care, dog

## Abstract

**Introduction:**

Periodontal disease is one of the most common health issues in dogs. However, disease is largely preventable by eliminating dental plaque, best achieved by daily tooth brushing. Unfortunately, owner adherence is low to the recommendation of daily tooth brushing in dogs.

**Objective:**

This study aimed to evaluate the impact of various communication strategies, traditional advice (TA) versus motivational interviewing (MI), and compare them to a control group receiving no additional communication (CG), on dog owners’ performance of dental home care and the oral health of their dogs.

**Methods:**

The study was conducted as a longitudinal clinical intervention study spanning 3 years, and involved 75 dog owners with young dogs who were randomly assigned to one of three groups: TA, MI, or CG. Intervention groups received annual telephone consultations based on their assigned methodology. A questionnaire was administered twice to all groups, and the dental health of the dogs was assessed at the study’s conclusion.

**Result:**

Tooth brushing frequency demonstrated a significant increase in the MI group compared to the CG group (*p* < 0.01), albeit with a relatively low occurrence of daily brushing among owners. Dental health assessment revealed a significantly lower plaque index in the MI group compared to the CG group (*p* < 0.05), and a lower calculus index in the TA group compared to the CG group (*p* < 0.01). No statistically significant differences were observed between the MI and TA groups in terms of dental health.

**Conclusion:**

Regular veterinary communication appears to have a positive influence on dog owner adherence to veterinary recommendations concerning dental care in dogs. Communication with veterinarians (MI and traditional advice) improved owner knowledge, attitude, and decreased frequency of not brushing. Although dental health parameters improved, the effect size was small, suggesting the complexity of adherence. Personalized calls to dog owners offer potential for dental health improvement, warranting further comparison of MI with traditional advice.

## Introduction

Periodontal disease, which includes gingivitis and periodontitis, is highly prevalent among dogs, affecting over 80% of those aged 3 and older (1–4). The disease is characterized by inflammation of the periodontal structures surrounding the teeth (5). Small and toy breeds are particularly susceptible, with the risk increasing as dogs age (6).

The initiating factor of periodontal disease is a bacterial biofilm on the teeth, known as dental plaque, but other factors, including host-response, also play a significant role in pathogenesis (6, 7). Dental plaque can mineralize into dental calculus, which is not pathologic in itself but facilitates bacterial adhesion and has thus been associated with inflammation and poor dental hygiene. However, the degree of calculus has been shown not to correlate with periodontitis (8, 9). One way to measure the degree of inflammation in a conscious animal is by using a thiol-detection strip. Thiol is a sulfuric compound produced by microbes and found in saliva, and it has been shown to correlate with the degree of inflammation (10). Left untreated, periodontal disease may ultimately result in tooth loss. However, the disease is largely preventable by eliminating or minimizing dental plaque, best achieved through daily tooth brushing (11), requiring good owner adherence (12).

Non-adherence to medical recommendations in people has been reported to be as high as 40%, and adherence to behavioral change even lower (13). Similar studies concerning veterinary health care advice are scarce but indicate equally low adherence (14). This includes adherence to dental home care recommendations, as demonstrated in a Swedish study conducted in 2017, where fewer than 4% of dog owners brushed their dog’s teeth daily, despite 43% stating that they had received a veterinarian’s recommendation to do so (15).

Effective communication is an important skill in veterinary practice. Veterinarians often need to motivate animal owners to implement behavioral changes to improve their animals’ health, such as regarding diet, medication, or home care (16). The way recommendations are communicated has been shown to affect the level of adherence, with client-centered communication being superior to the traditional paternalistic approach (17). Unfortunately, studies suggest that a paternalistic approach is still the most common way of communication /giving advice in veterinary settings (18–21). In this approach, the veterinarian adopts a more persuasive role, does most of the talking, and acts more of a guardian, whereas client-centered communication involves shared decision-making, where the client is more active, and the veterinarian’s role is more that of an advisor/counselor (17).

Motivational Interviewing MI is one such person-centered, evidence-based communication style increasingly used within human health care (22). In contrast to paternalism, MI is a collaboration centered counseling style aiming to awaken and strengthen a person’s inner motivation and commitment to change by exploring the client’s concerns, perspectives, and addressing ambivalence (23). Within MI, the clinician / interviewer helps the client resolve how they should act instead of telling the client what to do. The interviewer focuses on eliciting and reinforcing statements a client uses that favors change (i.e., change talk) and decreasing resistance to change by using communication techniques such as open questions, affirmations, reflections, and summaries (23). Studies have shown that this style of communication may be an effective way to promote and support behavior changes in clients (22, 24).

The aims of this study were to investigate how different communication approaches - traditional advice vs. motivational interviewing- compared to a control group receiving no additional information, affect dog owners’ performance of dental home care and the dental health of their dogs. Additional aims were to investigate how these communication approaches affect dog owners’ attitudes and knowledge toward dental home care for dogs, and their satisfaction with veterinary communication.

## Methods

### Study design

The study was designed as a long-term (3 years) clinical intervention study. Dogs were recruited from veterinary clinical records at one of two veterinary clinics/hospitals in Stockholm, Sweden (Anicura Gärdets Djurklinik or Anicura Albano Djursjukhus). The inclusion criteria were all dog owners who had visited the clinic/hospital for puppy vaccination at approximately 12 weeks, where the estimated adult weight of the dogs was <12 kg, due to the higher prevalence of periodontal disease in smaller dogs. All dog owners who met the inclusion criteria were contacted, and recruitment was discontinued upon reaching a sample size of 75 dog owners accepting the invitation. Dog owners were contacted between December 2019 and May 2020. The dogs included in the study were approximately 6 months old at the time, and written consent to participate was obtained.

The sample size estimate was based on the number of dog owners who brushed their dogs teeth after the intervention in a pilot-study (unpublished) and a previous questionnaire survey of brushing frequency in the general population (15). The dog owners were randomly (random number generator in Microsoft Excel) assigned to one of three groups: the MI group (*n* = 25), the traditional advice group (*n* = 25), or the control group (*n* = 25). The control group did not receive any telephone consultations/conversations after providing consent. All participants received information regarding the study’s purpose to investigate veterinary communication on dental home care in dogs.

**Figure 1:**
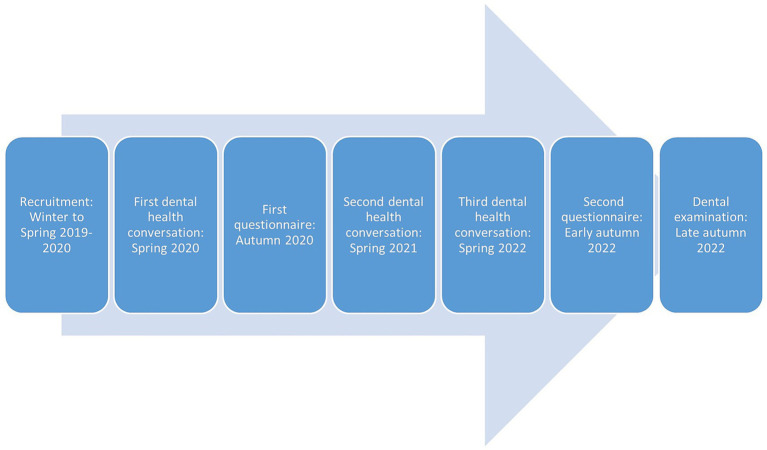
Timeline of the study.

The intervention groups, the traditional advice and the MI group, participated in telephone consultations/interviews regarding the dog’s dental health with a veterinarian specializing in veterinary dentistry (first author, KBE) on three occasions, approximately every 12 months until the end of the study. Dog owners were contacted via telephone, with a maximum of seven attempts made to reach each participant. In cases where there were difficulties in contacting participants, email and text messages were also used. The traditional advice group mainly received one-way (veterinarian talking most of the time) information on dental health and dental home care according to a semi-standardized protocol, with opportunities for questions. MI counseling was implemented according to MI methodology (23) as described further below, with information conveyed following MI recommendations (23). The timeline of the study is illustrated in Figure 1.

### Motivational Interviewing Treatment Integrity (MITI) 4.2.1

To validate the results and ensure treatment fidelity, the extent of Motivational Interviewing (MI) used during counseling sessions was assessed using the standardized protocol Motivational Interviewing Treatment Integrity 4.2.1 (MITI 4.2.1) (25). The MITI protocol comprises two main parts: global scores and behavior count (26).

The global scoring is based on coder assessment of technical components (Cultivating Change Talk, Softening Sustain Talk) and relationship components (Partnership, Empathy). These components are scored on a scale from one (least) to five (most) with three as default reference value. For behavior counts, the coder records the instances of 10 specific behaviors during the conversation. These behaviors include Giving Information, Persuasion, Persuasion with Permission, Asking Question, Simple Reflection, Complex Reflection, Affirming, Seeking Collaboration, Emphasizing Autonomy, and Confrontation. Among these behaviors, Persuasion and Confrontation are considered MI-non-adherent behaviors, while the remaining behaviors are more or less MI-adherent.

All initial conversations, both in the MI and traditional advice groups, were recorded to analyze the veterinarian’s conversational style. The audio files were submitted to MIC Lab AB, Stockholm^1^, for coding in accordance with MITI 4.2.1. The duration of the conversations was also documented.

### Questionnaire

All participating dog owners, including those in the control group, received a web-based Supplementary material (the questionnaire; Netigate AB), containing questions pertaining dog dental health, dental home care, and their experiences of the dental health conversation with the veterinarian The same questionnaire was administered after one and three years, as illustrated in Figure 1. During the second administration, the final item involved booking an appointment for a dental examination.

### Dental health examination

A clinical examination of dental health was conducted for all volunteering participating dogs at approximately 3 years of age, as depicted in Figure 1. The examination adhered to a standardized protocol (27, 28) (Table 1), and was performed by the same veterinarian (author KBE). During this examination, the teeth were evaluated for the degree of gingival inflammation (graded 0–3), plaque (graded 0–3) (27) and calculus (graded 0–3) (28), and the results of a thiol-detection test (OraStripdx, Pdx Biotech LLC, Washington, USA) (graded 0–5) (10) were recorded. Plaque assessment was carried out following the application of a plaque disclosing agent (Coloring Rondells Red, Nordenta, Enköping, Sweden), and photographs were taken for documentation purposes. To ensure that the assessor remained unaware of the dental home care regimen, questionnaires were not reviewed until after the examination.

**Table 1:** Dental health assessment protocol used in the study.

Gingival index (GI)	
0	no inflammation
1	mild inflammation, mild hyperemia
2	moderate inflammation, moderate hyperemia
3	severe inflammation, severe hyperemia, swelling, bleeding spontaneously, ulcerations
Plaque index (PI)	
0	no plaque
1	thin layer of plaque along the gingival edge
2	moderate layer of plaque and/or plaque in sulcus
3	abundant plaque and soft material in sulcus
Calculus index (CI)	
0	no calculus
1	supragingival or calculus that extends only slightly below the free gingival margin
2	moderate amount of supra- and/or subgingival calculus or only subgingival calculus
3	abundant supragingival and/or subgingival calculus

### Data analysis

MI methodology (MITI 4.2.1 coding) was analyzed using a two-sample, two-sided t-test, comparing all first conversations between the MI group and the traditional advice group. Dental health data (indices) and tooth brushing frequency were analyzed with one-way ANOVA, comparing the MI group, the traditional advice group and the control group. For main effect with *p* < 0.05, post-hoc pairwise tests were performed using t-tests with Tukey adjustment for a family of three estimates. ANOVA assumptions were assessed using residuals-*vs*-fitted and Q-Q plots. Results from *t*-tests and ANOVA are presented as mean values and *p*-values for pairwise comparisons. Associations between dental health indices, including thiol test, were investigated using polyserial correlation as part of the validation of the indices used in the study. All analyses were performed using R v 4.3.0.

## Results

As previously described, 75 dog owners with their dogs were recruited to the study [Supplementary material (list of participating dogs)].

### Conversation duration and treatment fidelity (MITI 4.2.1 protocol)

The number of conversations (reachable participants/ total participants (after dropouts) at the time) and the mean conversation length in the MI group were as follows:

- First conversation: 23/25 participants, with an average duration of 20 min and 28 s.
- Second conversation: 21/23 participants, lasting 12 min and 25 s.
- Third conversation: 19/22 participants, lasting10 min and 29 s.

In the traditional advice group, the numbers were as follows:

- First conversation: 24/25 participants, with an average duration of 13 min and 30 s.
- Second conversation: 21/24 participants, lasting 7 min and 27 s.
- Third conversation: 20/23 participants, lasting 7 min and 33 s.

**Figure 2:**
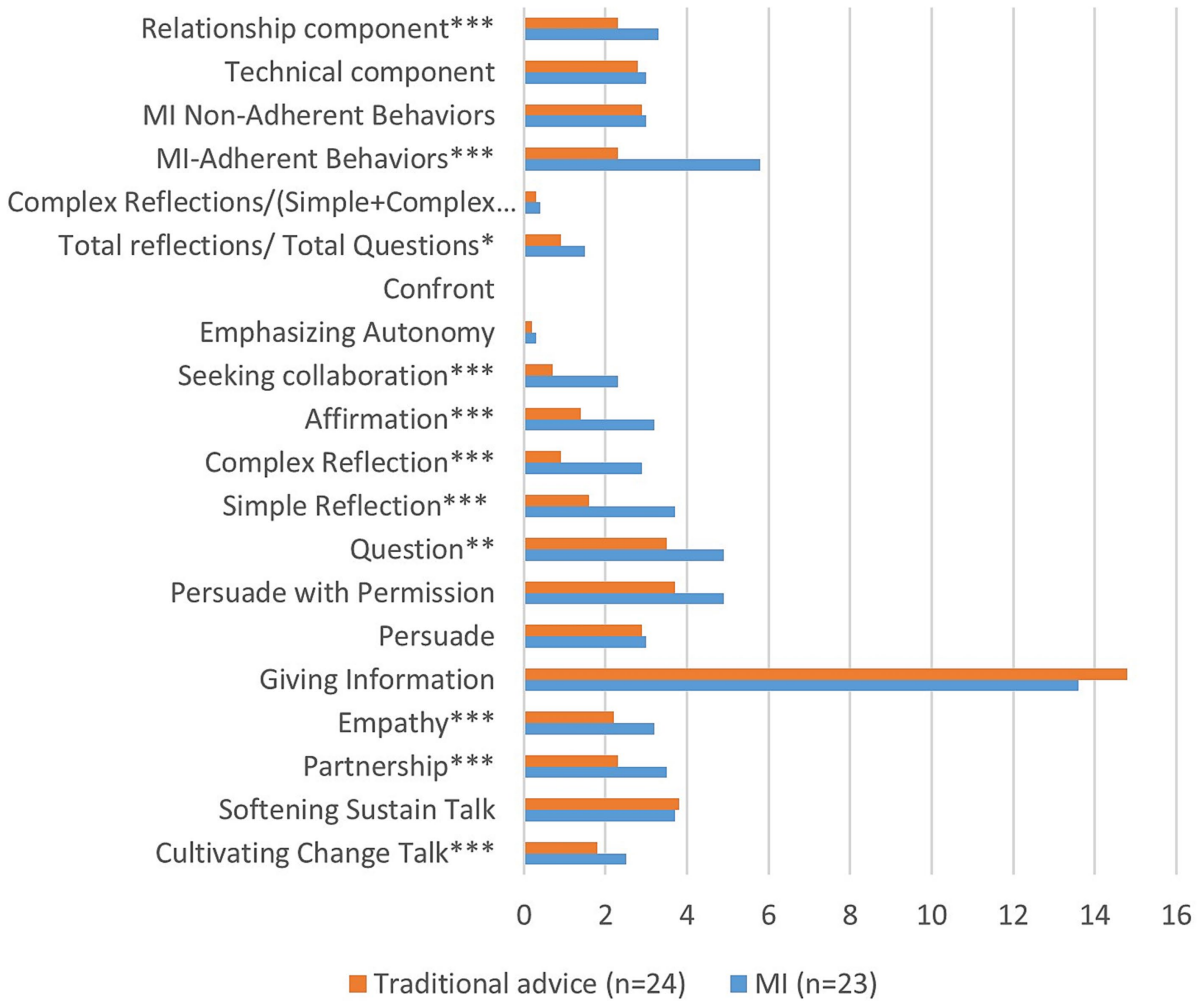
Result (means) of MITI 4.2.1 analysis of first conversations with MI group and traditional advice group, respectively. * = *p* < 0.05, ** = *p* < 0.01, *** = *p* < 0.001.

Treatment fidelity was ensured through MI-coding using the MITI 4.2.1 protocol. Statistically significant differences in communication methodology was shown between the MI group and the traditional advice group, as illustrated in Figure 2.

### Questionnaire survey: dog owners’ self-reported dental home care, attitudes toward oral care and dog’s dental health

The response rate for the first questionnaire survey, conducted when the dogs were approximately 1 year old, was 80% (60/75), and for the second survey, conducted when the dogs were around 3 years old was 63% (47/75). The median response time was 8 min and 54 s for the communication groups (MI and traditional advice) and 7 min and 50 s for the control group. In the first survey, there were 23 respondents in the MI group, 20 in the traditional advice group, and 17 in the control group. In the second survey, the numbers were 17 in the MI group, 16 in the traditional advice group, and 14 in the control group.

Table 2 provides an overview of key survey results. On the first occasion, 52–75% of respondents assessed their dogs’ dental health as “Very good,” but on the second occasion, this number had decreased to 21–38%. Another noteworthy contrast between the two surveys is the number of individuals who would consider brushing their dogs’ teeth, which decreased from the first to the second occasion in the MI group, while remaining approximately the same or slightly increasing in the two other groups. In addition, reported dental health deteriorated between the first and second occasions in all groups, including the occurrence of bleeding gums when brushing, halitosis, and the presence of dental calculus. It is worth noting that, at the second occasion, almost a third of dog owners did not know if their dog had dental calculus (Table 2).

**Table 2:** Questionnaire survey data on self-reported oral hygiene attitudes and behaviors collected at two time points: survey one (dogs approximately 1 year old) and survey two (dogs approximately 3 years old).

	Survey one (dogs approx 1 year)			Survey two (dogs approx 3 years)		
Item	MI (n_max_ = 23) % (n/n_tot_)	Traditional (n_max_ = 20) % (n/n_tot_)	Control (n_max_ = 17) % (n/n_tot_)	MI (n_max_ = 17) % (n/n_tot_)	Traditional (n_max_ = 16) % (n/n_tot_)	Control (n_max_ = 14) % (n/n_tot_)
How would you appraise your dog’s dental health?						
*Very good*	52% (12/23)	75% (15/20)	59% (10/17)	29% (5/17)	38% (6/16)	21% (3/14)
*Fairly good*	30% (7/23)	15% (3/20)	29% (5/17)	47% (8/17)	31% (5/16)	57% (8/14)
*Neither good nor bad*	0% (0/23)	0% (0/20)	6% (1/17)	6% (1/17)	13% (2/16)	21% (3/14)
*Fairly poor*	0% (0/23)	0% (0/20)	0% (0/17)	0% (0/17)	0% (0/16)	0% (0/14)
*Very poor*	0% (0/23)	0% (0/20)	0% (0/17)	0% (0/17)	0% (0/16)	0% (0/14)
*Do not know*	17% (4/23)	10% (2/20)	6% (1/17)	18% (3/17)	19% (3/16)	0% (0/14)
Would you consider brushing your dog’s teeth daily? *(Yes or Maybe)*	95% (18/19)	84% (16/19)	59% (10/17)	64% (9/14)	88% (14/16)	64% (9/14)
Dog’s dental health (reported by DO)						
*Bleeding gum (sometimes, often, or always) Question only to the ones who brush.*	5% (1/22)	19% (3/16)	23% (3/13)	56% (9/16)	29% (4/14)	33% (3/9)
*Halitosis (sometimes, often, or always)*	42% (8/19)	37% (7/19)	35% (6/17)	59% (10/17)	53% (9/17)	64% (9/14)
*Dental calculus present (a little, moderately, or a lot)*	11% (2/19)	0% (0/19)	24% (4/17)	53% (9/17)	44% (7/16)	64% (9/14)
*Dental calculus (Do not know)*	37% (7/19)	21% (4/19)	24% (4/17)	29% (5/17)	31% (5/16)	29% (4/14)
Perceived importance of tooth brushing in dog *Response: Very important*	59% (13/22)	55% (11/20)	53% (8/15)	31% (5/16)	75% (12/16)	38% (5/13)
Started (or tried) to brush when recommended by the veterinarian in the study, but then discontinued.	26% (6/23)	20% (4/20)	NA	41% (7/17)	25% (4/16)	NA
Started to brush when recommended by the veterinarian in the study, still brushes.	26% (6/23)	35% (7/20)	NA	41% (7/17)	31% (5/16)	NA
Satisfaction with veterinary communication on a scale of 0–10 *(mean)*	8.7	9.1	NA	8.5	8.8	NA

The number of respondents for each question (n_tot_) is provided in the result-box, and these counts may vary from the maximum number of respondents (n_max_) due to internal data loss. DO denotes the dog owner.

#### Knowledge of dental cleaning methods

In another survey question, respondents were asked to rank four different methods for dental cleaning. In survey two, 76% of dog owners in the MI group and 69% in the traditional advice group identified tooth brushing as the most effective method, while in the control group, this percentage was 50%.

#### Utilization of dental cleaning methods

In survey two, eight dog owners reported using textiles at least once a week to clean their dog’s teeth: 3 out of 17 in the MI group, 5 out of 16 in the traditional advice group, and none in the control group. Additionally, 15 respondents reported using dental chews (specifically designed for dental health) at least once a week: 8 out of 17 in the MI group, 4 out of 15 in the traditional advice group, and 3 out of 14 in the control group.

#### Previous dental procedure

Five dogs had previously undergone anesthesia for dental cleaning on the second survey occasion: two in the traditional advice group, two in the MI group, and one in the control group.

#### Use of dental scalers

In survey two, respondents were asked if a dental scaler had been used by them or someone else (e.g., groomer or breeder) to remove their dog’s dental calculus. Three respondents in the MI group and four in the control group answered affirmatively, while none in the traditional advice group reported using a dental scaler.

#### Tooth brushing frequency

**Figure 3:**
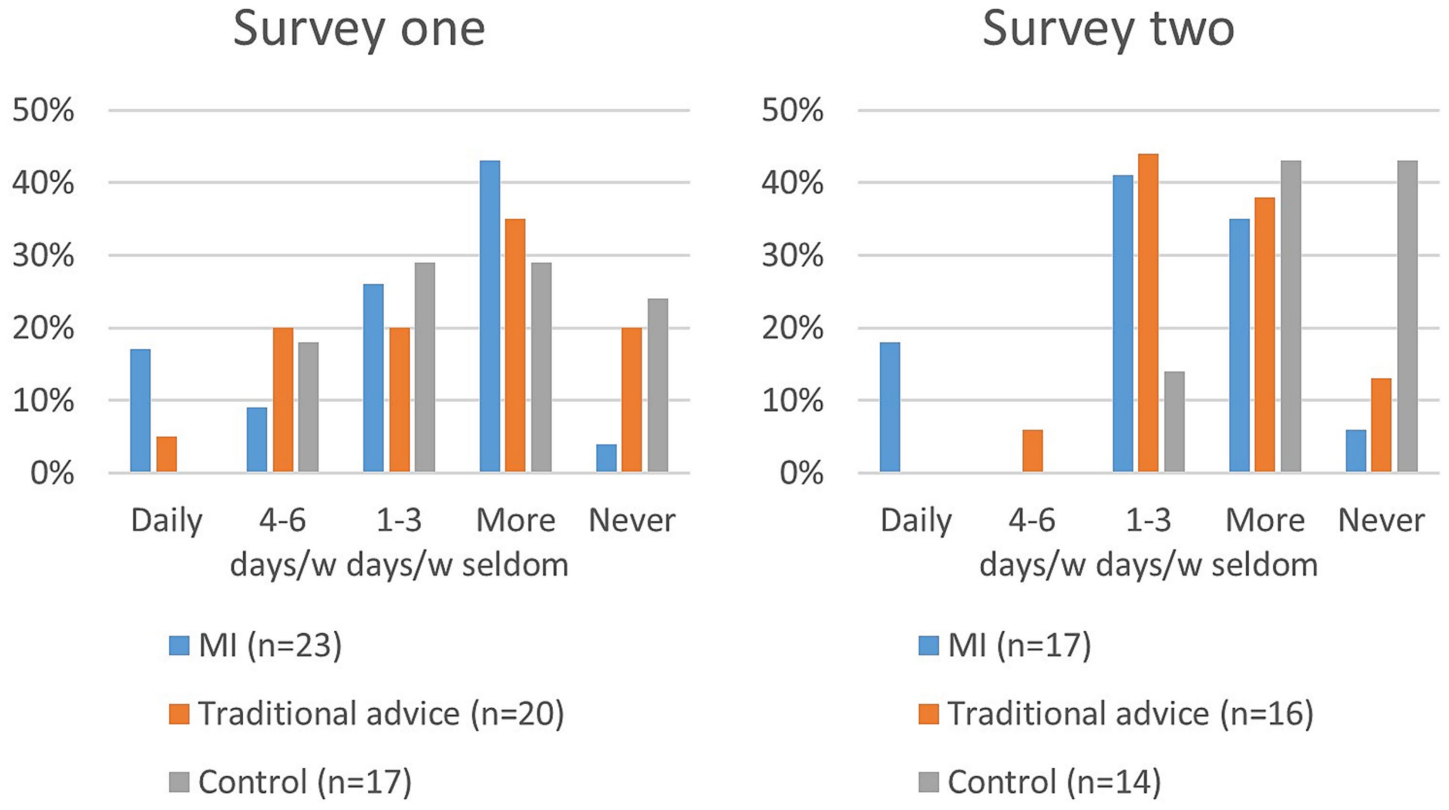
How often, in the last month, have you brushed your dog’s teeth with a toothbrush? (Survey one and two).

A majority of dog owners brushed their dog’s teeth less frequently than once a week. Tooth brushing frequency showed a decrease from the first to the second survey, as depicted in Figure 3.

**Figure 4:**
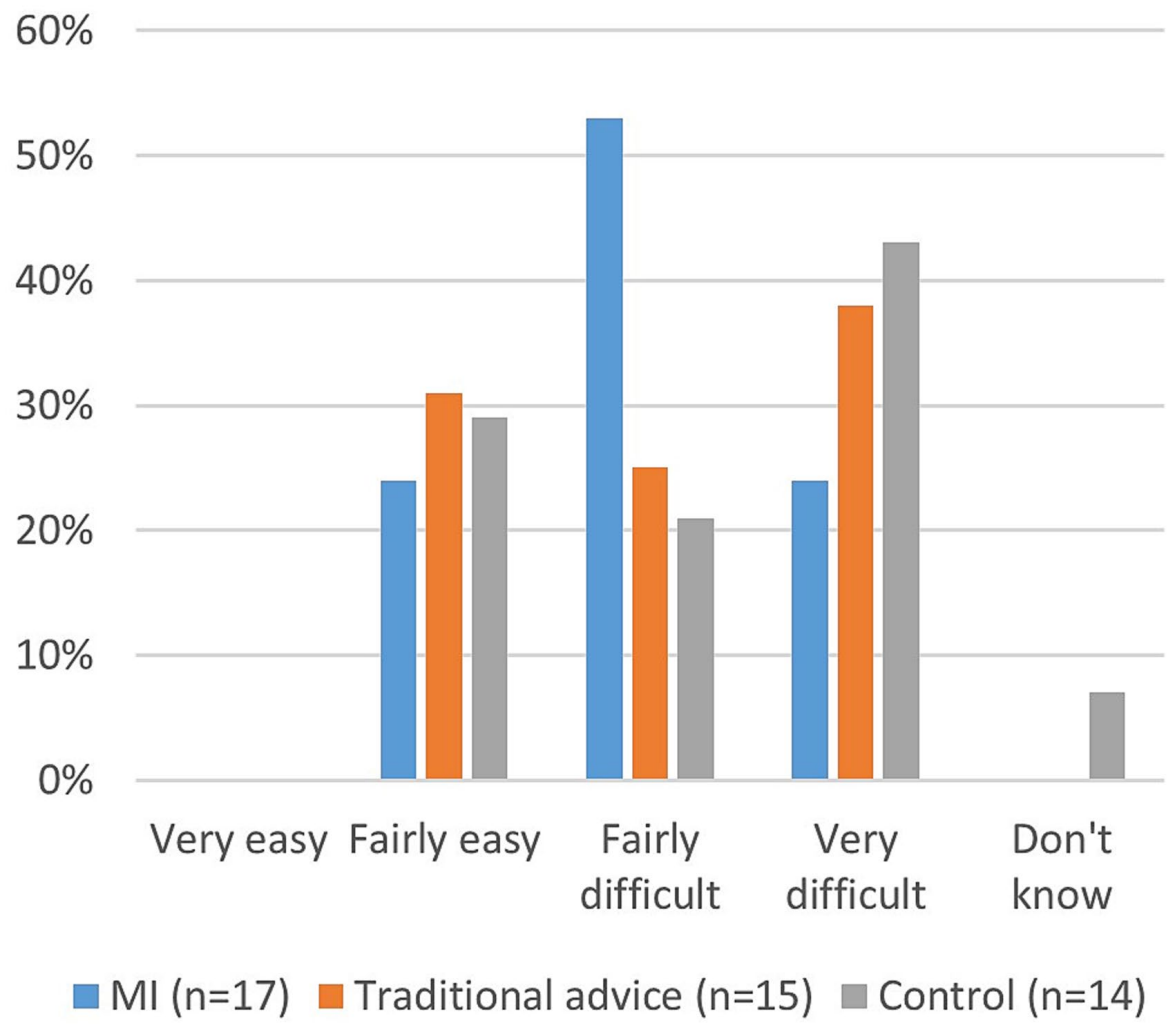
How easy or difficult is it for you to brush all of your dog’s teeth? (Survey two).

A majority of dog owners experienced difficulties with tooth brushing, as reported in survey two (Figure 4).

Tooth brushing frequency was significantly higher in the MI group compared to the control group (*p* = 0.003) in survey two; no other group comparisons were significant.

#### Satisfaction with communication

Satisfaction with the communication in the conversation groups was measured on a scale of 0–10, where 10 depicted maximum satisfaction. In survey two, the mean satisfaction score in the MI group was 8.5, while in the traditional advice group, it was 8.8 (Table 2).

### Quality assurance of dental health indices

Results of associations between dental health indices and thiol test are presented in Table 3. The thiol test exhibited moderate correlations with GI, PI, and CI. The calculus index showed a high correlation with the gingival index (Table 3).

**Table 3:** Index correlation polyserial: O.7–0.9 is considered highly correlated, 0.5–0.7 moderately correlated.

	GI	PI	CI	Thiol test
GI	1	0.18	0.73	0.51
PI		1	0.49	0.59
CI			1	0.54
Thiol test				1

GI denotes gingival index, PI denotes plaque index, and CI denotes calculus index.

### Dental health

**Figure 5:**
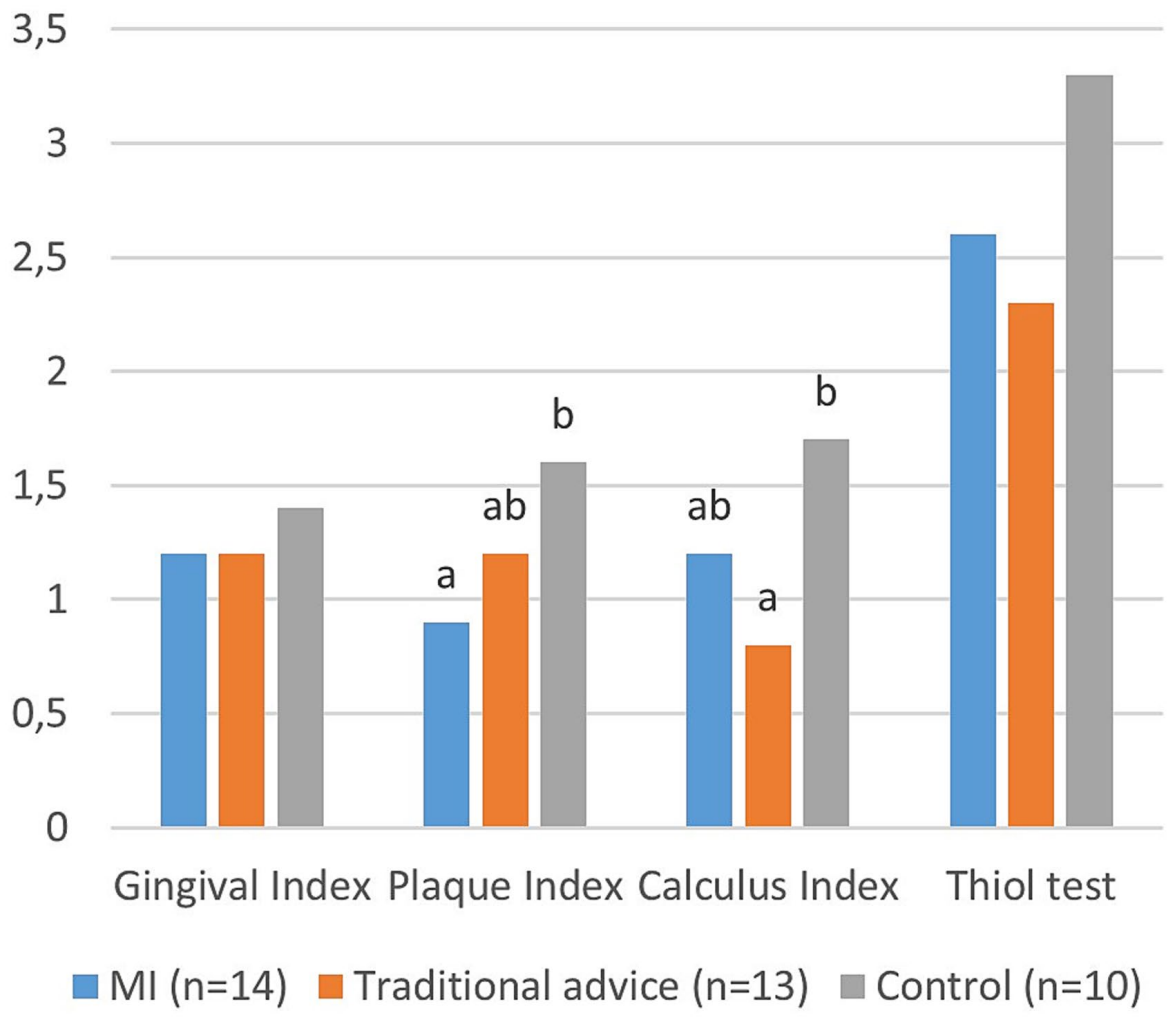
Dental Health Assessment results: Mean values for the gingival index (0–3), plaque index (0–3), calculus index (0–3), and thiol-detection test (0–5) are depicted. The gingival index (*p* = 0.483) and thiol test (*p* = 0.127) did not differ statistically between groups. However, the plaque index (*p* = 0.019) and calculus index (*p* = 0.003) showed significant differences between groups. Groups with different letters are significantly different at *p* < 0.05.

A total of 37 out of 69 remaining dog owners accepted the invitation for a dental clinical examination at the end of the study, comprising 14 from the MI-group, 13 from the traditional advice group, and 10 from the control group. The overall drop-out rate for the clinical examination was thus 51% (38/75 dog owners).The results of the dental health examination are presented in Figure 5. Data analysis revealed a significantly lower plaque index for the MI-group compared to the control group (*p* = 0.019). The calculus index was significantly lower for the traditional advice group compared to the control group (*p* = 0.003). There was no significant difference in dental health parameters between the two intervention groups (Figure 5).

**Figure 6:**
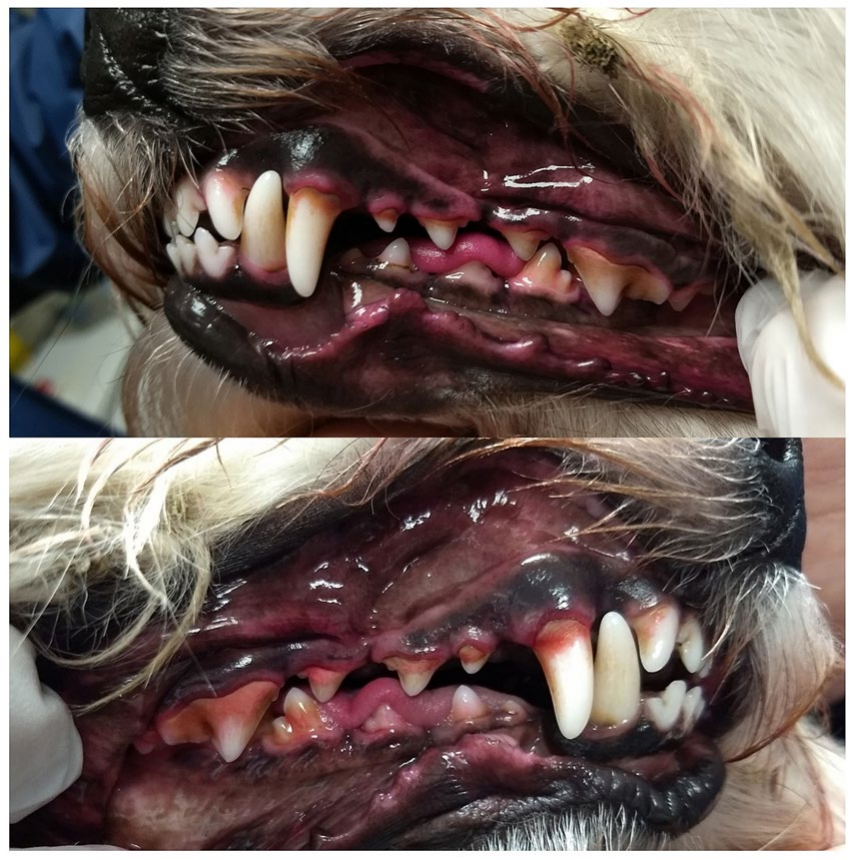
An example of a dental assessment result for a dog in the study: the calculus index is 2, the gingival index is 2, and the plaque index is 1. In the accompanying picture, after using a disclosing agent, the plaque appears pink. The owner reported brushing once a week, with difficulty. In the questionnaire, the owner assessed the level of calculus as ‘none’ (MI-group).

An example of a dental assessment is shown in Figure 6.

All but four dogs, one of which had recently undergone professional dental cleaning and one had extractions of teeth 108 and 208 due to fractures exhibited some degree of dental calculus upon examination. These four dogs with calculus index zero were in the traditional advice group.

Out of the 37 dogs examined, 12 dog owners were unaware of whether their dogs had dental calculus. Among these 12, 11 dogs were found to have dental calculus upon examination, with an even distribution between the groups (Figure 6). Additionally, among the seven dog owners who reported that their dogs did not have any dental calculus, five of the dogs were assessed as having dental calculus upon examination, which was also observed in all groups. This illustrates a lack of awareness among owners.

Examination after coloring the plaque revealed that all dogs had some degree of plaque. Although no overt signs of periodontitis, such as gingival recession or mobile teeth, were observed during the examination, periodontal probing was not performed, thus periodontitis cannot be excluded.

## Discussion

Significant differences in dental health, with lower plaque index observed in dogs whose owners received MI intervention and lower calculus index in dogs whose owners received traditional advice compared to those who did not receive any information (control group), underscore the importance of regular follow-ups and providing information. However, no significant differences in dental health were found between the MI and traditional advice groups. Several findings from both questionnaire surveys and the dental examination were consistent with earlier studies (15, 29, 30). This consistency occurred despite dog owners in the study receiving repeated messages emphasizing the importance of daily oral care to maintain good oral health, highlighting the complexity of communication interventions and behavioral changes.

Satisfaction with the veterinary conversations, regardless of the group, was consistently high, suggesting additional benefits for the clinic in maintaining regular communication with dog owners.

### Questionnaire survey

The deterioration in perceived dental health between the first and second survey, as indicated by dog owner’ assessments of their dog’s dental health, along with increased reports of occasional gum bleeding, halitosis, and dental calculus, was expected. Infrequent tooth brushing is known to result in deteriorating dental health over time, even in relatively young dogs (1). Bleeding gums are a sign of persistent gingivitis, which is unsurprising given the low frequency of tooth brushing in the study. Halitosis is most commonly associated with dental disease (5), including gingivitis, and increase accordingly. Regarding dental calculus, previous studies have suggested a prevalence of 61–100% (1, 4, 9, 31). Dental calculus is also an indicator of poor dental hygiene and was reported by 53% of dog owners, but it was detected in almost all three-year-old dogs during the examination, increasing the evidence that dental calculus is under-reported by dog owners.

For comparison, we analyzed previously collected national data (15, 29), yielding a group of 3,789 individual three-year-old dogs weighing less than 15 kg (unpublished data). The percentage of halitosis in the present study (55%) was comparable to the national data (51%). In the national data, occasional bleeding was reported by 31%, whereas it was reported by 46% in the present study. The larger percentage in this study might be attributed to more owners in our study attempting tooth brushing and therefore noticing bleeding. Additionally, the reported prevalence of calculus was somewhat higher in our study (53%) compared to the national data (39%), which may indicate greater awareness among the owners in our study. However, as mentioned, underreporting of dental calculus by dog owners was extensive in our study.

### Tooth brushing

From the first to the second survey, there was a decline in the number of dog owners in the MI group who considered daily brushing, while the numbers in the two other groups slightly increased. The reason for this decline is unknown, but tooth brushing frequency was highest in the MI group, possibly leading to a higher proportion of failures. We hypothesize that motivation is initially high in all groups when owning a puppy or young dog and after receiving a recommendation from the veterinarian to brush their dog’s teeth. However, motivation may decrease when difficulties are encountered and when the dog is perceived to have good dental health. Conversely, motivation may increase when signs such as halitosis and dental calculus become evident.

To have a significant impact on gingival health, effective brushing should be performed daily or almost daily (11). Surprisingly and disappointingly, only three owners were brushing daily at the end of the study. All of these were however in the MI group. Only one dog owner brushed 4–6 times a week (traditional advice group). The corresponding percentages are approximately the same as in the population of three-year-old dogs under 15 kg (unpublished data), despite the intervention. We suggest that the presence of disease serves as a stronger motivator to start and maintain brushing (32). However, prophylactic measures are preferable to treating manifest disease. The present study has shown that even with early information, supposedly motivated puppy owners, and regular follow-ups, tooth brushing frequency remains low. Nonetheless, considerably more dog owners in the control group answered that they never brush (Figure 3), showing a positive effect of the intervention. Although the clinical relevance of infrequent brushing is low, it may mean that owners who occasionally brush are more likely to increase their frequency if they encounter dental problems.

When comparing different methods for dental cleaning, more owners in the traditional advice group (75%) than in the other groups knew that tooth brushing was the most effective method. This indicates a higher awareness due to the clear recommendation and information provided. Moreover, all seven owners who reported using a dental scaler to remove calculus on their dog were in the MI or control group. Although the number of such cases was low, it may reflect the fact that the use of a dental scaler on an awake animal was more strongly discouraged in the traditional advice group, suggesting the potential benefit of admonition.

A majority of owners encountered difficulties while brushing their dogs’ teeth, such as the dog chewing, wriggling, and resisting. This indicates that the conversations in the study were insufficient in addressing these encountered difficulties. Such information was conveyed during the interview in connection to the dental examinations, but very few written answers were received despite explicit requests for them. To adhere to prophylactic oral health care recommendations, regardless of communication method, training both the dog and the owner in handling the dog’s mouth is necessary to achieve a cooperative dog and successfully perform recommended tooth brushing.

The present study suggests that yearly phone communication alone is insufficient for increasing daily brushing. Tooth brushing frequency increased, as indicated by the rise in “seldom-brushing,” but the overall impact was limited. Pet owners should ideally receive information about their dogs’ dental health and preventive dental care during wellness examinations at the veterinary clinic. To maximize adherence to recommendations, the most effective interventions have shown to be integrating multiple components (33). We suggest that client-centered communication and education, both theoretical and practical, verbal and written, preferably including pictures and films, as well as demonstrations of active dental home care, during regular follow-ups would be beneficial for increasing adherence to dental home care recommendations. This approach is used within human dentistry today with regular visits to the dental hygienist to discuss dental home care and prophylactic measures, as well as regular dental examinations.

### Dental health

Significant improvements in dental health were observed in the intervention groups compared to the control group, specifically in terms of plaque (MI-group) and calculus (traditional advice group). However, no significant differences were observed in gingivitis or the thiol detection test. Additionally, nearly all dogs displayed some degree of dental calculus during the examination. This outcome was unsurprising given the infrequent practice of daily brushing among the dogs in the study.

Thiol, a volatile sulfuric compound, has previously been shown to correlate to the degree of inflammation (10, 34), and our study aligns with these findings. The lack of significance in the gingival index and thiol levels may be attributed to the overall low brushing frequency, which falls short of preventing or treating gingivitis, consistent with prior research emphasizing the necessity of daily or near-daily brushing for optimal dental health (11).

It is worth noting that even among the four dogs brushed daily or 4–6 times a week, gingivitis, plaque, and calculus were still present. This underscores the importance of not only the frequency but also the quality and effectiveness of brushing. Additionally, there was no apparent correlation between tooth brushing frequency and dental health indices, possibly due to the low number of dogs brushed daily. Consequently, results should be interpreted with caution. Furthermore, post-plaque-staining photographs revealed some degree of plaque in all dogs in the study, including those who had recently brushed. This finding aligns with a meta-review indicating an average plaque reduction of only 42% following tooth brushing in humans (35), emphasizing the significance of proper brushing technique in conjunction with increased frequency.

Dental calculus facilitates adhesion of dental plaque and is associated with inflammation (36). It is noteworthy that many dog owners across all groups were unaware of whether their dog had dental calculus, or answered that there dog did not have dental calculus, when nearly all of these dogs exhibited dental calculus upon examination. Even though it is not clear whether this is due to a lack of knowledge or difficulties inspecting, these findings underscore the need for enhanced owner education. In our study, communication occurred primarily through telephone calls, precluding the opportunity to physically demonstrate what dental calculus looks like or to provide techniques, tips, and tricks for tooth brushing. In future communication plans, it is advisable to include discussions on dental calculus alongside periodontal disease and tooth brushing habits.

As the dogs in the study were not examined under anesthesia, periodontal probing was not conducted. While the dogs did not display signs of gingival recession or loose teeth, it is possible that they already had clinical attachment loss (periodontitis), as several studies have reported clinical attachment loss in dogs under 3 years old (1, 37). The degree of periodontal disease is often underestimated when examining conscious animals without sedation or anesthesia (12), underscoring the likelihood that some of the dogs in the study already had periodontitis.

### Communication

Adherence is multifaceted and complex and MI has been suggested as an important component of this important puzzle (38). However, in this study, MI demonstrated no significant difference compared to traditional advice concerning dental health parameters or tooth brushing frequency (4–7 times a week). Nevertheless, distinctions were observed between the communication groups and the control group, highlighting the benefits of the intervention.

Satisfaction with communication within the conversation groups was notably high, with an average rating of 8.5–9.1 on a scale ranging from 0 to 10. Although assessed only descriptively, this indicates that owners were overall highly satisfied with the veterinary communication in both groups. Given the likelihood of variations in communication styles among different counselors, it is important to note that in this study, all conversations were conducted by the same veterinarian, author KBE. This choice was made to minimize inter-counselor bias. Conversations were also subjected to MITI-coding to ensure fidelity to MI and to confirm differences between treatment groups. KBE, an experienced professional in dental home care advice situations, might have effectively conveyed information in a manner that resonated with both MI and traditional advice groups. The involvement of another counselor might have yielded different results.

Satisfaction with communication holds significance beyond mere adherence to recommendations; it may also enhance the likelihood of clients returning for future visits and promote the clinic to other pet owners. In subsequent veterinary encounters, satisfaction and trust can facilitate treatments. Notably, MI has previously been shown to increase client satisfaction in medical settings (39).

A substantial portion of dog owners, ranging from 20 to 41%, reported that they initially attempted to brush their dogs’ teeth upon the veterinarian’s recommendation, but later discontinued the practice. This aligns with findings from a larger 2017 study in Sweden where 26% of participants ceased brushing after being advised by a veterinarian to do so (15). This suggests that the limited number of conversations in both communication groups might not have been sufficient to address perceived difficulties with brushing. It also raises the possibility that an intervention solely through telephone calls may be insufficient, implying the need for more frequent follow-ups, potentially involving veterinary visits to provide support in overcoming obstacles.

Previous studies have demonstrated that paternalistic communication is prevalent among veterinarians (20, 21), despite the recommendation for client-centered communication (17). In this study, the individual who conducted the conversations, KBE, ensured method fidelity through the MITI protocol. Nevertheless, prior knowledge of client-centered communication could have played a role in the limited differentiation observed among certain parameters between the conversation groups. Moreover, KBE possessed training in MI but limited practical experience with the method. To yield more representative data, future research should involve larger studies with greater diversity and the inclusion of multiple counselors.

All tools and aids that assist in communicating dental disease to pet owners hold value, and a thiol detection test may be one such tool (12, 40). Other potential methods to improve conscious examination and communication with pet owners could include plaque staining and photographs of the oral cavity. Although these techniques were employed in the present study and communicated during the final dental examination visit, the study’s design did not permit the evaluation of whether these interventions increased compliance with tooth brushing recommendations. The integration of these tools and aids, along with tooth brushing, is greatly facilitated by early training of dogs to accept these procedures. Therefore, training should be incorporated into future dental health programs.

### Methodological considerations

The study initially had an excellent response rate, with only 18 out of all contacted dog owners declining to participate. However, as the study progressed, this enthusiasm waned. The response rate for the second questionnaire and the number of owners participating in the free dental examination were unexpectedly low, despite the study’s short questionnaire, free veterinary examination, and the topic’s relevance to dog owners. The drop-out reasons remain unclear, but it is possible that the motivation for health promotion and preventive care was not as strong as when dealing with diagnosed diseases. This may have contributed to the difficulty in reaching owners during follow-up calls. Regular post-visit follow-up calls from the veterinary clinic, instead of a research study, could potentially yield different outcomes. Furthermore, increasing the frequency of follow-ups, such as scheduling them bi-annually, might lead to even more favorable outcomes.

Numerous studies have highlighted the challenge of investigating Motivational Interviewing (MI) despite its promising outcomes (22, 24, 38). Methodological challenges, encompassing issues with investigating treatment fidelity, have been identified, and the method exhibits inconclusive evidence (41–43). However, using the MITI-protocol to confirm treatment fidelity is an advantage in MI studies. In the present study, MI-coding using the MITI 4.2.1 protocol demonstrated significant differences in communication methodology, particularly within the relational components, between the MI-group and the traditional advice group. However, some parameters did not show statistically significant differences, suggesting limited effective distinctions between communication groups. Additionally, the fact that many conversations in this study were shorter than the recommended 20 min may partly explain the absence of significant differences in outcomes between the communication groups. The longer time required for MI in this study may indicate difficulties in implementing MI in a veterinarian’s everyday practice. However, trained general practitioners report that motivational interviewing takes about the same amount of time as traditional approaches to giving advice (44).

The same person administered intervention and assessed outcome. To minimize impact of this lack of blinding, the last survey was not assessed until after the clinical examination. The risk of attrition- and dropout-bias should also be noted, e.g., that the most interested dog owners were the ones remaining in the study. This may be indicated by the larger dropout in the control group.

Further analysis of recorded material has the potential to offer a deeper understanding of the motivational factors influencing pet owners’ decisions regarding dog dental care and tooth brushing.

## Conclusion

Communication with the veterinarian, both Motivational Interviewing (MI) and traditional advice, resulted in increased knowledge and a more positive attitude toward tooth brushing, as well as a reduced frequency of owners who never brushed their dogs’ teeth. Two dental health parameters were significantly better in the communication groups. However, the effect size was small, possibly reflecting the complexity of adherence to veterinary recommendations. Nevertheless, personalized calls to dog owners to discuss and follow up on dental home care have the potential to improve dental health in dogs. MI, as a tool for improving adherence to veterinary dental home care advice, requires further research, including studies involving multiple experienced counselors, to determine if this method is superior to traditional advice.

## Data availability statement

The original contributions presented in the study are included in the article/Supplementary material, further inquiries can be directed to the corresponding author.

## Ethics statement

Ethical approval was not required for the studies involving humans according to the the Swedish Ethical Review Authority, Uppsala. The studies were conducted in accordance with the local legislation and institutional requirements. The participants provided their written informed consent to participate in this study. Ethical approval was not required for the studies involving animals in accordance with the local legislation and institutional requirements because dental examination was considered non-invasive. Written informed consent was obtained from the owners for the participation of their animals in this study.

## Author contributions

KE: Conceptualization, Data curation, Investigation, Visualization, Writing – original draft. BJ: Writing – review & editing. KA: Writing – review & editing. AP: Conceptualization, Funding acquisition, Supervision, Writing – review & editing.

## Footnotes

^1^ https://miclab.se
