# Supplementary material for: Long-term effects of motivational interviewing vs. traditional counseling on dog owners’ adherence to veterinary dental home care: a three-year follow-up study
Source: Front Vet Sci. 2024 Feb 26;11:1296618. doi: 10.3389/fvets.2024.1296618 (PMC11002956; doi:10.3389/fvets.2024.1296618)
Supplement: Supplementary file 1 [file Data_Sheet_1.PDF]

Supplemental material belonging to the manuscript:

Long-Term Effects of Motivational Interviewing vs. Traditional Counseling on Dog Owners' Adherence to Veterinary Dental Home Care: A Three-Year Follow-Up Study

Karolina Brunius Enlund, Birgitta Jönsson, Kajsa H. Abrahamsson, Ann Pettersson

---

A translation of the original Swedish survey into English by Karolina Brunius Enlund.

---

1. Approximately how much does your dog weigh?

- ☐ 0 - 2,9kg
- ☐ 3 - 5,9kg
- ☐ 6 - 9,9kg
- ☐ 10 - 14,9 kg
- ☐ 15 - 19,9 kg
- ☐ 20 - 29,9 kg
- ☐ 30 kg or more
- ☐ Don´t know

2. How would you appraise your dog's general health?

- ☐ Very bad
- ☐ Fairly bad
- ☐ Neither good nor bad
- ☐ Fairly good
- ☐ Very good
- ☐ Don't know

3. How would you appraise your dog's dental health ?

- ☐ Very bad
- ☐ Fairly bad
- ☐ Neither good nor bad
- ☐ Fairly good
- ☐ Very good
- ☐ Don't know / Can not judge

4. How important is it for you that your dog has good dental health?

- ☐ Not at all important
- ☐ Of minor importance
- ☐ Fairly important
- ☐ Very important
- ☐ Don't know

5. What do you consider to be important to good dental health in the dog?

	Not at all important	Of minor importance	Fairly important	Very important	Don't know
**Good general health**	☐	☐	☐	☐	☐
**Special dog food that according to the manufacturer benefits dental health**	☐	☐	☐	☐	☐
**Chewingbones, e.g. rawhide, bully sticks, pig ears or pig tails**	☐	☐	☐	☐	☐
**Tooth brushing**	☐	☐	☐	☐	☐
**Dental cleaning with textiles (e.g. fingercloth, microfiber, cloth or gauze)**	☐	☐	☐	☐	☐
**The dogs breed / heredity**	☐	☐	☐	☐	☐
**Special dental chews that according to the manufacturer benefits dental health**	☐	☐	☐	☐	☐
**Dog toothpaste**	☐	☐	☐	☐	☐

6. Rank the following four ways to care for your dog's teeth, from most to least effective.

- Tooth brushing
- Special dental chews that according to the manufacturer benefits dental health
- Chewingbones, e.g. rawhide, bully sticks, pig ears or pig tails
- Special dog food that according to the manufacturer benefits dental health

7. How often in the last month have you brushed your dog's teeth with a toothbrush?

- ☐ Daily
- ☐ 4-6 days / week
- ☐ 1-3 days / week
- ☐ More seldom / single occasion
- ☐ Never
- ☐ Don't know

8. How often in the last month have you cleaned your dog's teeth with textiles (eg fingercloth, microfiber, fabric or gauze)?

- ☐ Daily
- ☐ 4-6 days / week
- ☐ 1-3 days / week
- ☐ More seldom / single occasion
- ☐ Never
- ☐ Don't know

***9.* Would you consider brushing your dog's teeth daily?**

- ☐ Yes
- ☐ Maybe
- ☐ No
- ☐ Do not want to answer

***10.* When you clean your dog's teeth at home, does the gum bleed?**

- ☐ No, never
- ☐ Yes, sometimes
- ☐ Yes, often
- ☐ Yes, always
- ☐ Don't know / Do not want to answer

11. How easy or difficult is it for you to brush all of your dog's teeth?

- ☐ Very easy
- ☐ Fairly easy
- ☐ Fairly difficult
- ☐ Very difficult
- ☐ Don't know

If you experience any difficulties, which ones?_____________________________________

12. How often in the last month has your dog chewed / used any of the following:

	Daily	4-6 days / week	1-3 days / week	More seldom / single occasion	Never	Don't know
**Special dental chews that according to the manufacturer benefits dental health**	☐	☐	☐	☐	☐	☐
**Dog toothpaste**	☐	☐	☐	☐	☐	☐
**Special dog food that according to the manufacturer benefits dental health**	☐	☐	☐	☐	☐	☐

13. Has an instrument for scraping dental tartar been used by yourself or someone else (e.g. groomer or breeder) to remove the dog's tartar?
Note: This does not include tartar removal at a veterinary clinic.

☐ No
☐ Yes, once
☐ Yes, several times
☐ Don't know

14. Has your dog been anesthetized at a veterinary clinic to clean the teeth / remove dental tartar?

☐ No
☐ Yes, once
☐ Yes, several times
☐ Don't know

15. Has your dog had problems with gum disease or loose teeth?
(Does not apply to puppy teeth)

☐ No
☐ Yes, the dog has had to extract teeth at the veterinary clinic and / or lost teeth at home
☐ Yes, but the dog has not had to extract any teeth
☐ Don't know

16. Does your dog have bad breath?

☐ No, never
☐ Yes, sometimes
☐ Yes, often
☐ Yes, always
☐ Don't know

17. Does your dog have dental tartar at the moment?

- ☐ No
- ☐ Yes, a little
- ☐ Yes, a moderate amount
- ☐ Yes, a lot
- ☐ Don't know

The following only to communication groups:

18. **When you received the recommendation from the veterinarian in the study: Did the information lead you to initiate brushing / cleaning your dog's teeth?** ***(only visible to respondents who answered that they had been recommended by a veterinary clinic on question 8)***

- ☐ I brushed / cleaned before I received the recommendation
- ☐ Yes, I brush / clean still
- ☐ Yes, I started (or tried) to brush / clean but stopped later
- ☐ No
- ☐ Don't know

19. Overall, how do you think the communication between you and the veterinarian in the study worked?

0=Very poorly
1
2
3
4
5
6
7
8
9
10=Very good

20. How did you experience the amount of dental care information you received from the veterinarian in the study?

Too little information
Right amount of information
Too much information
Don’t know

21. Overall, how have you experienced participating in the study so far?

0=Very displeased
1
2
3
4
5
6
7
8
9
10=Very pleased

22. If you have anything additional that you would like to present, you are welcome to write it here.
