# Supplementary material for: Long-term effects of motivational interviewing vs. traditional counseling on dog owners’ adherence to veterinary dental home care: a three-year follow-up study
Source: Front Vet Sci. 2024 Feb 26;11:1296618. doi: 10.3389/fvets.2024.1296618 (PMC11002956; doi:10.3389/fvets.2024.1296618)
Supplement: Supplementary file 2 [file Data_Sheet_2.PDF]

Supplementary

Long-Term Effects of Motivational Interviewing vs. Traditional Counseling on Dog Owners' Adherence to Veterinary Dental Home Care: A Three-Year Follow-Up Study

*TA denotes Traditional Advice group, MI denotes Motivational Interviewing group, and C denotes control group

Group and number*	Sex	Breed
TA 1	F	Lagotto romagnolo
TA 2	F	Havanese
TA 3	F	Dachshund standard, long-haired
TA 4	F	Chihuahua
TA 5	M	French Bulldogg
TA 6	M	Mixed breed (Miniature poodle)
TA 7	M	Miniature Poodle
TA 8	M	Mixed breed (Cockerpoo)
TA 9	F	Miniature Bull Terrier
TA 10	M	English Cocker Spaniel
TA 11	F	Brussels Griffon
TA 12	F	Miniature Schnauzer
TA 13	F	Pomeranian
TA 14	F	Mixed breed (Poodle/Maltese)
TA 15	M	Miniature Poodle
TA 16	M	Miniature Schnauzer
TA 17	F	Mixed breed (Pomeranian/Chihuahua)
TA 18	F	Danish-Swedish Farmdog
TA 19	M	Pomeranian
TA 20	F	English Cocker Spaniel
TA 21	F	Lagotto romagnolo
TA 22	F	Mixed breed (Cockerpoo)
TA 23	M	Shetland Sheepdog
TA 24	F	Mixed breed (Cockerpoo)
TA 25	F	Italian Greyhound
MI 1	F	Mixed breed (Havanese/Poodle)
MI 2	F	Norfolk Terrier
MI 3	F	Dachshund standard, smooth-haired
MI 4	M	English Cocker Spaniel
MI 5	M	Italian Greyhound
MI 6	M	Havanese
MI 7	M	Mixed breed (Havanese)
MI 8	M	Japanese Spitz
MI 9	M	Miniature Poodle

MI 10	M	Bichon havanais
MI 11	M	Mixed breed (Havanese/ Poodle)
MI 12	M	English Cocker Spaniel
MI 13	M	Japanese Spitz
MI 14	M	Bedlington Terrier
MI 15	M	Havanese
MI 16	F	Mixed breed (Cavalier King Charles Spaniel / Pug)
MI 17	F	Mixed breed (Cockerpoo)
MI 18	M	Cavalier King Charles Spaniel
MI 19	F	Havanese
MI 20	F	French Bulldogg
MI 21	F	Mixed breed (Pug, Dachshound, English Cocker Spaniel)
MI 22	M	Labradoodle australian
MI 23	F	Mixed breed (Cavapoo)
MI 24	F	Chihuahua
MI 25	M	Welsh Corgi Pembroke
C1	F	English Cocker Spaniel
C2	F	Miniature Poodle
C3	F	Mixed breed (Bichon Frise/Miniature Poodle)
C4	F	Shetland Sheepdog
C5	F	Havanese
C6	F	Mixed breed (Cockerpoo)
C7	F	Miniature Poodle
C8	M	Nederlandse Kooikerhondje
C9	F	Fox Terrier
C10	F	Mixed breed (Cavapoo)
C11	M	Beagle
C12	F	Mixed breed (Cavapoo)
C13	F	Mixed breed (Bichon Frise/Miniature Poodle)
C14	F	Welch Corgi Cardigan
C15	M	Mixed breed (Cavalier King Charles Spaniel/English Cocker Spaniel)
C16	F	English Cocker Spaniel
C17	F	Dachshund miniature, smooth-haired
C18	F	Dachshund miniature, smooth-haired
C19	F	Whippet
C20	M	Medium size Poodle
C21	M	Mixed breed (Cockerpoo)
C22	M	Mixed breed (Bichon Frise/Shih Tzu)
C23	M	English Cocker Spaniel
C24	M	Mixed breed (Cockerpoo)
C25	M	Mixed breed (Prazský krysarík/Pomeranian)
